# 

**Published:** 1830-01

**Authors:** 


### LONDON

### MEDICAL AND PHYSICAL

### JOURNAL.

---

EDITED BY

JOHN NORTH, ESQ. F.L.S.

MEMBER OF THE ROYAL COLLEGE OF SURGEONS,

AND OF THE MEDICAL AND CHIRURGICAL SOCIETY OF LONDON.

---

(VOL. LXIV.)

NEW SERIES, VOL. VIII.

Et quoniam variant morbi, variabimus artes;

Mille mali species, mille salutis erunt.

---

45596

LONDON:

JOHN SOUTER, 73, ST. PAUL'S CHURCH-YARD,

AND TO BE HAD OF ALL THE MEDICAL BOOKSELLERS.

1830.

W1
LO 31

J. AND C. ADLARD, PRINTERS,

BARTHOLOMEW CLOSE.

### MONTHLY LIST OF MEDICAL BOOKS.

[*Medical Works cannot be entered on this List except a copy be sent for the purpose*; *the titles of Books having frequently been transmitted to us, as published, which have not appeared for weeks, or even months, after.*]

Sketches of Intellectual and Moral Relations. By DANIEL PRING, M.D. Member of the Royal College of Surgeons, London.—8vo. pp. 466. Longman and Co. London, 1829.

Elements of Physics; or, Natural Philosophy, General and Medical, explained independently of Technical Mathematics. In two vols. Vol. II. Part I. comprehending the Subjects of Heat and Light. By NEIL ARNOTT, M.D. of the Royal College of Physicians.—8vo. pp. 320. Longman and Co. and Underwood, London, 1829.

A Manual for Students who are preparing for Examination at Apothecaries' Hall. By JOHN STEGGALL, M.D. M.R.C.S. &c. FOURTH EDITION, with considerable Additions and Improvements.—Royal 18mo. pp. 333, with Plates. Highley, London, 1830.

The student, who has already passed through his various classes, will derive much advantage from looking over this volume. By studying the numerous questions and answers it contains upon Chemistry, Materia Medica, Anatomy, Physiology, Nosology, Practice of Medicine, Midwifery, &c. he will impress upon his mind the information he has previously received. It will not be a less useful assistant to him who has yet to enter upon his studies: it will prepare him to follow, and properly to appreciate, the doctrines of his teachers, with much more facility and pleasure to himself than if he entered upon his task without having first gained the elementary principles of his profession. The last regulations issued from Apothecaries' Hall are affixed to the work.

A Practical Treatise on the anti-Asthmatic Properties of the Bladder-podded Lobelia (Lobelia Inflata, Linn.); with Directions for the Exhibition of the Preparations of it, which have succeeded in the Practice of the most eminent Physicians. With Instructions as to Diet, Exercise, &c., and Remarks on the supposed Varieties of the Disease. To which is added, an Account of the Chirargyta Herb, lately introduced as a Remedy for Nervous and Gouty Indigestion, Morbid Sensibility of the Stomach, and Obstructions of the Liver, &c. By RICHARD REECE, M.D. Fellow of the Royal College of Surgeons, &c.—Ridgway, London.

A Practical Treatise on Diseases of the Genitals of the Male; with a Preliminary Essay on the History, Nature, and general Treatment of Lues Venerea. By JOHN MADDOX TITLEY, M.D.—8vo. pp. 403. Herbert, London, 1829.

Hospital Facts and Observations, illustrative of the Efficacy of the new Remedies, Strychnia, Brucia, Acetate of Morphia, Veratria, Iodine, &c. in several Morbid Conditions of the System. With a comparative View of the Treatment of Chorea, and some Cases of Diabetes. A Report on the Efficacy of Sulphureous Fumigations in Diseases of the Skin, Chronic Rheumatism, &c. By JAMES LOMAX BARDSLEY, M.D. Physician to the Manchester Infirmary.—8vo. pp. 223. Burgess and Hill, 1829.

The Muscles of the Human Body, describing their Origin, Insertion, and Use. Arranged in four Tables. For the use of Students.—Burgess and Hill, London, 1829.

For the moderate price of one shilling, the student has here presented to him a clear and correct account of all the muscles of the human body. If their circulation is in proportion to their utility, these tables will have an extensive sale.

Researches principally relative to the Morbid and Curative Effects of Loss of Blood. By MARSHALL HALL, M.D. F.R.S.E.—8vo. pp. 303. Seeley and Sons, London, 1830.

*Communications have been received from* Dr. GRIFFIN, Mr. D. GRIFFIN, Dr. ASHLEY GAITSKELL, Dr. HUTCHINSON, Mr. WALLER, *and* Mr. COX.

